# Sensitivity to change and minimal clinically important difference of the angioedema control test

**DOI:** 10.1002/clt2.12295

**Published:** 2023-09-01

**Authors:** Lauré M. Fijen, Carolina Vera, Thomas Buttgereit, Hanna Bonnekoh, Marcus Maurer, Markus Magerl, Karsten Weller

**Affiliations:** ^1^ Department of Vascular Medicine Amsterdam Cardiovascular Sciences Amsterdam UMC University of Amsterdam Amsterdam The Netherlands; ^2^ Institute of Allergology Charité ‐ Universitätsmedizin Berlin corporate member of Freie Universität Berlin and Humboldt‐Universität zu Berlin Berlin Germany; ^3^ Fraunhofer Institute for Translational Medicine and Pharmacology ITMP Immunology and Allergology Berlin Germany

**Keywords:** angioedema, disease control, minimal clinically important difference (MCID)7, patient‐reported outcome measures (PROMs), sensitivity to change

## Abstract

**Background:**

The Angioedema Control Test (AECT) is a patient‐reported outcome measure developed and validated for the assessment of disease control in patients with recurrent angioedema. Its sensitivity to change and minimal clinically important difference (MCID) have hitherto not been established.

**Methods:**

Patients with recurrent angioedema due to chronic spontaneous urticaria, hereditary angioedema, or acquired C1‐inhibitor deficiency were repeatedly asked to complete the AECT along with the Angioedema Quality of Life Questionnaire (AE‐QoL), Dermatology Life Quality Index (DLQI), and anchors for disease control and whether treatment was sufficient during routine care visits. The sensitivity to the change of the AECT was determined by correlating changes in its scores over time with changes in the applied anchors. The MCID was determined using anchor‐based and distributional criterion‐based approaches.

**Results:**

Eighty‐six cases were used for this analysis. Changes in AECT scores correlated well with AE‐QoL changes (but less with changes in the DLQI) as well as other applied anchors, demonstrating its sensitivity to change. The MCID was found to be three points for improvement of angioedema control. The available number of cases with meaningful deterioration in our dataset was too low to reach a definite conclusion on the MCID for deterioration of angioedema control.

**Conclusion:**

The AECT is a valuable tool to assess changes in disease control in patients with recurrent angioedema over time. The lowest AECT score change that reflects a meaningful improvement of disease control to patients (MCID) is three points.

## 1 INTRODUCTION

Angioedema is defined by a localised and self‐limiting swelling of the subcutaneous and/or submucosal tissue due to a temporary increase of vascular permeability. In recurrent angioedema, swellings occur episodically and can affect several locations of the body, that is, the tongue, extremities, abdomen or upper respiratory tract.^1^ Most cases of recurrent angioedema are either mast cell‐mediated (e.g. in patients with chronic urticaria) or bradykinin‐mediated (e.g. in patients with hereditary angioedema due to C1‐inhibitor deficiency [HAE‐C1INH] or acquired C1‐inhibitor deficiency [AAE‐C1INH]).

The high burden of recurrent angioedema is caused by its unpredictable, painful, disfiguring, disabling, and sometimes even life‐threatening clinical manifestation. Angioedema attacks affect daily activities, social relations, and cause high rates of absenteeism and presenteeism.^2^, ^3^, ^4^ Several prophylactic and acute treatment options are available for most types of recurrent angioedema. They differ in efficacy, administration route, and side effects but are all aimed at improving disease control, since curation is (currently) not possible.^5^, ^6^ Complete disease control is the treatment goal in mast cell‐mediated and bradykinin‐mediated recurrent angioedema.^6^, ^7^, ^8^, ^9^

Given the unpredictability and day‐to‐day fluctuation of symptoms,^10^ it is difficult to establish disease activity, disease burden, and treatment response based on clinical signs and symptoms during routine care visits.^11^ To effectively treat patients with recurrent angioedema, it is important to have valid and reliable tools to capture the actual disease status.^12^, ^13^ Therefore, several patient‐reported outcome measures (PROMs) have been developed for patients with recurrent angioedema,^14^, ^15^, ^16^ including the Angioedema Control Test (AECT).^6^, ^8^ The AECT has been developed and validated to assess disease control in patients with recurrent angioedema.^17^, ^18^ Previous studies have shown that it is well suited for routine practice,^19^, ^20^ clinical research,^21^, ^22^, ^23^, ^24^, ^25^ and therapeutic trials^26^ given its retrospective approach, brevity, and simple scoring. The AECT has excellent internal consistency and test‐retest reliability, as well as high convergent validity and good known‐groups validity.^18^, ^27^ The cut‐off scores for identifying patients with well‐controlled disease and poorly controlled disease have been established at ≥10 and <10, respectively.^18^, ^27^

For both clinical practice and trials, it is imperative to know the ability of the AECT to determine changes over time, for example, before and after treatment adjustment. However, as of yet, this property of the AECT has not been investigated. Apart from dichotomising AECT scores in poor or good angioedema control, the interpretation of AECT score changes is difficult because it is currently unclear which score changes are meaningful to patients. In other words, the minimal clinically important difference (MCID), that is, the smallest change that patients would identify as a noticeable and meaningful improvement, of the AECT is unknown. To address these gaps of knowledge, the current study aimed to determine the sensitivity to change and MCID of the AECT.

## 2 METHODS

### 2.1 Patient population

Consecutive German‐speaking patients with recurrent angioedema aged 12 years or older treated at the Angioedema Center of Reference and Excellence (ACARE, https://acare‐network.com)^28^ of the Institute of Allergology of the Charité—Universitätsmedizin Berlin were invited to participate. Informed consent was obtained from all individual participants included in the study. The participants were asked to complete the German version of the AECT along with other PROMs during several successive routine care visits. This study was approved by the ethics committee of the Charité—Universitätsmedizin Berlin (EA4/020/20).

### 2.2 Patient‐reported outcome measures

#### 2.2.1 Angioedema control test

The AECT is an angioedema‐specific, valid, and reliable questionnaire that assesses disease control. It can be used in all patients with recurrent angioedema, that is, mast cell‐mediated (e.g. chronic spontaneous urticaria) and bradykinin‐mediated (e.g. HAE‐C1INH) angioedema. Two separate versions, with a recall period of 4 weeks and 3 months are available. The present study used the version with a 4 week recall period.^17^ The AECT consists of four questions with five answer options each (scored with 0–4 points). Accordingly, AECT scores range from 0 to 16 points, with 16 points indicating complete disease control. Cut‐off scores for well versus poorly controlled disease have been established at ≥10 and <10, respectively.^18^, ^27^ Participants were asked to answer the AECT at baseline and at a follow‐up visit.

#### 2.2.2 Patients' self‐assessment of global disease control, change in global disease control, and treatment sufficiency

Along with the AECT, all patients were asked to self‐rate their global angioedema control during the past 4 weeks on a 5‐point Likert scale (Pat‐GA‐control, answer options: ‘completely controlled’, ‘well controlled’, ‘moderately controlled’, ‘hardly controlled’, ‘not at all controlled’). In addition, they indicated if their angioedema treatment in the past 4 weeks was ‘sufficient’ or ‘not sufficient’. At the follow‐up visit, patients were also asked to self‐rate the global change in angioedema control in comparison to the last time they filled out the AECT on a 7‐point Likert scale (Pat‐GA‐control‐change, answer options: ‘improvement to complete control’, ‘clearly better controlled’, ‘slightly better controlled’, ‘no change in control’, ‘slightly worse controlled’, ‘clearly worse controlled’, ‘deterioration to complete lack of control’). The treating physicians of the patients were asked to fill out these questions from their perspective as well (Phy‐GA‐control and Phy‐GA‐control‐change).

#### 2.2.3 Quality of life measures

**FIGURE 1:**
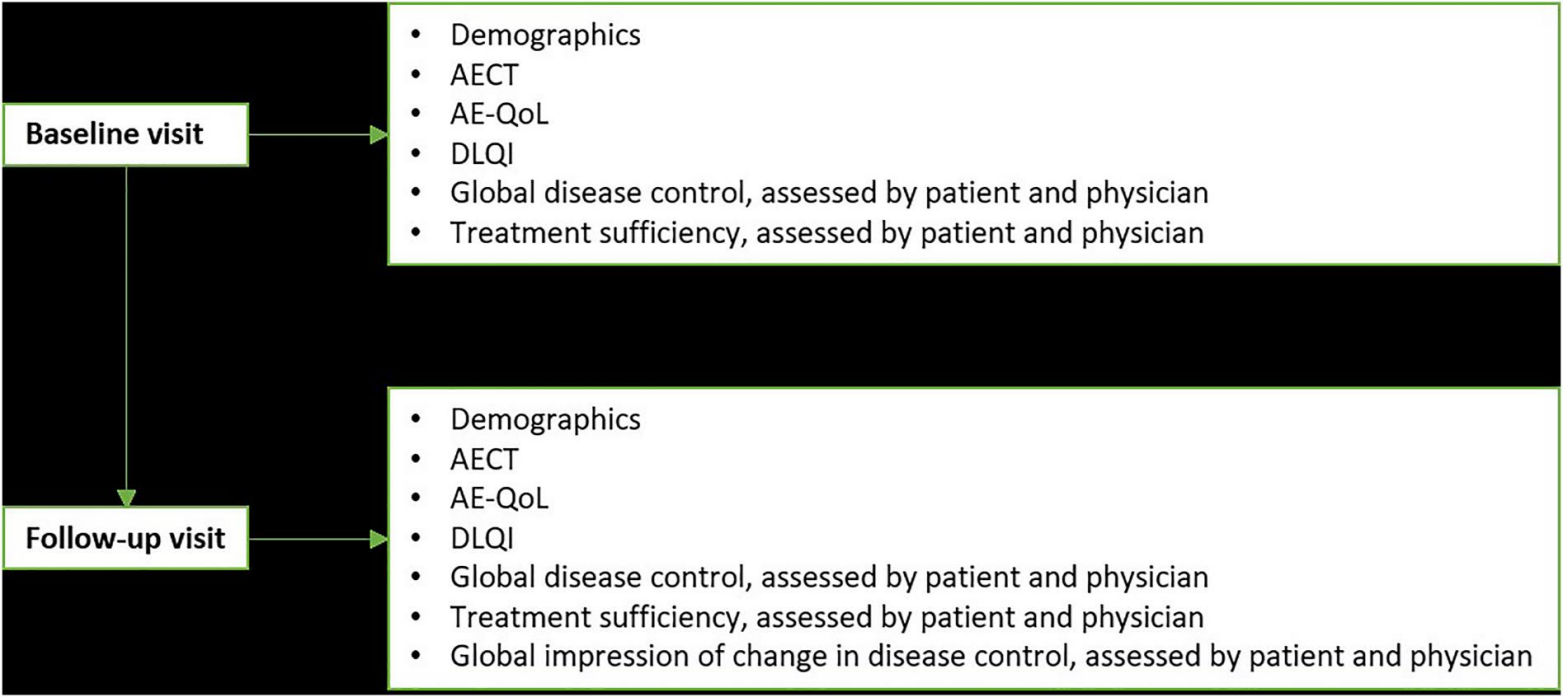
Study flow chart. At the baseline and follow‐up visit demographics, the AECT and several anchors were obtained. AECT, Angioedema Control Test; AE‐QoL, Angioedema Quality of Life Questionnaire; DLQI, Dermatology Life Quality Index.

The Angioedema Quality of Life Questionnaire (AE‐QoL) is an angioedema‐specific, validated health‐related quality of life (QoL) measure with a recall period of 4 weeks. It contains 17 questions from which a total score on a 0–100 scale can be computed.^15^, ^29^ The Dermatology Life Quality Index (DLQI) is a health‐related QoL measure for dermatological disorders with a recall period of 7 days. It contains 10 questions from which a sum score on a 0–30 scale can be computed.^30^ Higher scores for the AE‐QoL and DLQI are both indicative of a higher QoL impairment. The patients filled out both questionnaires along with the AECT at both visits. Figure 1 shows the study flow diagram including the information obtained and anchors used at the baseline and follow‐up visits. Patients who visited the out‐patient clinic more than two times during the course of this study could fill‐out additional follow‐up visit questionnaires.

### 2.3 Data analysis

#### 2.3.1 Sensitivity to change

Sensitivity to change is the ability of a PROM to detect change over time in the patient's disease status, regardless of whether this change is clinically relevant or meaningful. To assess the sensitivity to the change of the AECT, we computed the rank correlation coefficient (Spearman's *rho*) for AECT total and individual question score changes between two different time points with changes in the AE‐QoL, DLQI, Pat‐GA‐control, Phy‐GA‐control and treatment sufficiency. The rank correlation coefficient was also calculated for the AECT score changes with the patients' and physicians' global impression of change in disease control, that is, the Pat‐GA‐control‐change and Phy‐GA‐control‐change, respectively, at the follow‐up visit.

#### 2.3.2 Responsiveness

Responsiveness is the ability of an instrument to determine meaningful changes in the patient's disease status over time. It is commonly reported through the minimal clinically important difference (MCID). A change equal to or higher than the MCID can be considered a meaningful change. To determine the MCID of the AECT, we applied anchor‐based and distributional criterion‐based approaches, as described previously.^31^, ^32^ The anchor‐based approaches were applied by computing the mean intra‐individual differences of AECT total and individual question scores between assessments with different Pat‐GA‐control ratings (defined as a change of one step, e.g. from no at all controlled to hardly controlled, or from well controlled to moderately controlled) and AE‐QoL change (defined as a change of one step. A one‐step change was defined as an AE‐QoL change of 6 to <12 points as the MCID of the AE‐QoL is 6 points.^29^ A two‐step change was defined as an AE‐QoL change of 12 to <18 points). In addition, the intra‐individual variation of AECT total and individual question scores in case of stable disease (unchanged PAT‐GA‐control or AE‐QoL) were analysed. For the use of Pat‐GA‐control and AE‐QoL for these responsiveness analyses, the correlation (Spearman's *rho*) of their changes and AECT score changes should be 0.5 or higher. Finally, the Pat‐GA‐control was used to perform a receiver operating characteristic (ROC) curve analysis to identify the best cut‐off point for clinically meaningful changes in the AECT total score. For this analysis, patients were categorised as subjects with a change in angioedema control (defined as at least one‐step change in their global angioedema control rating) and subjects without a change in angioedema control. Likewise, ROC curve analysis was performed with patients divided into groups based on change in AE‐QoL scores (e.g. at least one‐step change, which was defined as an AE‐QoL change of at least 6 points). The ROC cut‐off point was chosen by getting the smallest sum of percentages of false positive and false negative classifications ([1‐sensitivity] + [1‐specificity]).^33^ The distributional criterion approach to calculate the MCID indirectly is based on the finding that one‐half of the standard deviation (SD) of an instrument's results may represent a good approximation of its MCID.^32^ Accordingly, the SD of all baseline AECT total scores was computed and subsequently divided by two. Another distribution‐based approach is one standard error of the mean (SEM), as this may represent an approximation of the MCID as well.^34^

#### 2.3.3 Sensitivity analysis

If patients filled out more than two rounds of questionnaires, these additional cases were included in the analyses. As a sensitivity analysis, all analyses were repeated with one pair of questionnaires from unique individuals.

#### 2.3.4 Statistical analysis

All statistical analyses were conducted in R version 4.0.3.^35^ The statistical methods applied are described in the respective methods and/or results sections of this manuscript. *p* ≤ 0.05 was considered statistically significant. Missing data were not imputed. Data from eight cases concerning patients' assessment of global angioedema control and whether or not treatment was sufficient were removed due to implausibility.

## 3 RESULTS

### 3.1 Demographics

In total, 86 cases (66 individual patients, average number of AECTs completed per patient: 2.5) were included, with a mean ± SD age of 50.5 ± 17.1 years (Table 1). Of the 66 participants, 39 (59%) had bradykinin‐mediated angioedema and 24 (36%) had mast cell‐mediated angioedema. HAE‐C1INH was the most frequent diagnosis (41%), and 73% of all cases were women. The median AECT score (interquartile range) at baseline was 10 (6–13) and 13 (7–16) at follow‐up.

**TABLE 1:** Patient characteristics.

Type of angioedema	All	Chronic urticaria with angioedema	Isolated angioedema	HAE type 1 or 2	HAE‐nC1INH	AAE‐C1INH	Unknown
*n*	66	9	15	27	1	11	3
Sex (%)							
Female	48 (72.7)	6 (66.7)	9 (60.0)	21 (77.8)	1 (100.0)	8 (72.7)	3 (100.0)
Male	18 (27.3)	3 (33.3)	6 (40.0)	6 (22.2)	0 (0.0)	3 (27.3)	0 (0.0)
Baseline							
Age (mean (SD))	50.50 (17.11)	48.78 (19.72)	54.13 (17.33)	44.78 (15.57)	46.00 (NA)	71.91 (9.69)	63.33 (5.77)
Antihistamine (%)	10 (15.2)	5 (55.6)	5 (53.3)	0 (0.0)	0 (0.0)	0 (0.0)	0 (0.0)
Steroids (%)	6 (9.1)	1 (11.1)	5 (33.3)	0 (0.0)	0 (0.0)	0 (0.0)	0 (0.0)
Omalizumab (%)	6 (9.1)	2 (22.2)	2 (13.3)	0 (0.0)	0 (0.0)	0 (0.0)	2 (66.7)
Icatibant (%)	22 (33.3)	0 (0.0)	2 (13.3)	12 (44.4)	1 (100.0)	7 (63.6)	0 (0.0)
C1‐inhibitor on demand (%)	10 (15.2)	0 (0.0)	0 (0.0)	8 (29.6)	0 (0.0)	2 (18.2)	0 (0.0)
C1‐inhibitor prophylactic (%)	5 (7.6)	0 (0.0)	0 (0.0)	5 (18.5)	0 (0.0)	0 (0.0)	0 (0.0)
Lanadelumab (%)	13 (19.7)	0 (0.0)	0 (0.0)	10 (37.0)	0 (0.0)	3 (27.3)	0 (0.0)
Other (%)	3 (4.5)	2 (22.2)	1 (6.7)	0 (0.0)	0 (0.0)	0 (0.0)	0 (0.0)
No treatment (%)	7 (10.6)	2 (22.2)	3 (20.0)	0 (0.0)	0 (0.0)	1 (9.1)	1 (33.3)
Unknown treatment (%)	1 (1.2)	0 (0.0)	0 (0.0)	0 (0.0)	0 (0.0)	1 (9.1)	0 (0.0)
Follow‐up							
Antihistamine (%)	15 (22.7)	6 (75.0)	8 (53.3)	0 (0.0)	0 (0.0)	1 (9.1)	0 (0.0)
Steroids (%)	5 (7.6)	0 (0.0)	3 (20.0)	0 (0.0)	0 (0.0)	0 (0.0)	2 (66.7)
Omalizumab (%)	12 (18.2)	5 (55.6)	5 (33.3)	0 (0.0)	0 (0.0)	0 (0.0)	2 (66.7)
Icatibant (%)	21 (31.8)	0 (0.0)	1 (6.7)	12 (44.4)	1 (100.0)	7 (63.6)	0 (0.0)
C1‐inhibitor on demand (%)	10 (15.5)	0 (0.0)	0 (0.0)	8 (29.6)	0 (0.0)	2 (18.2)	0 (0.0)
C1‐inhibitor prophylactic (%)	5 (7.6)	0 (0.0)	0 (0.0)	4 (14.8)	0 (0.0)	1 (8.3)	0 (0.0)
Lanadelumab (%)	16 (24.2)	0 (0.0)	0 (0.0)	12 (44.4)	0 (0.0)	4 (36.4)	0 (0.0)
Other (%)	3 (4.5)	1 (11.1)	1 (6.7)	0 (0.0)	0 (0.0)	1 (9.1)	0 (0.0)
No treatment (%)	12 (18.2)	1 (11.1)	3 (20.0)	5 (18.5)	0 (0.0)	3 (27.3)	0 (0.0)
Unknown treatment (%)	0 (0.0)	0 (0.0)	0 (0.0)	0 (0.0)	0 (0.0)	0 (0.0)	0 (0.0)

Abbreviations: AAE‐C1INH, acquired angioedema with C1‐inhibitor deficiency; HAE, hereditary angioedema; HAE‐nC1INH, hereditary angioedema with normal C1‐inhibitor; SD, standard deviation.

### 3.2 The AECT shows high sensitivity to change

The AECT total score change over time correlated significantly with the patients' and physicians' global impression of change in disease control, assessed by Pat‐GA‐control‐change and Phy‐GA‐control‐change, respectively. AECT changes were also correlated with those of the Pat‐GA‐control, Phy‐GA‐control, AE‐QoL, and DLQI scores as well as with treatment sufficiency (Table 2). The strength of the correlation of AECT changes and anchor results was high (*r* > 0.5), except for the DLQI (*r* = 0.3) and Pat‐GA‐control‐change (*r* = 0.3).

**TABLE 2:** Correlations of changes in patients' and physicians' assessed global disease control, AE‐QoL, DLQI, and treatment sufficiency with AECT total and individual scores.

	AECT total score change	AECT Q1 change	AECT Q2 change	AECT Q3 change	AECT Q4 change
Global disease control change, assessed by patient (change in pat‐GA‐control)^a^ (95% CI)	0.82 (0.73 to 0.91)	0.64 (0.48 to 0.80)	0.79 (0.68 to 0.90)	0.61 (0.46 to 0.76)	0.86 (0.78 to 0.95)
	*p* < 0.001	*p* < 0.001	*p* < 0.001	*p* < 0.001	*p* < 0.001
AE‐QoL change^b^ (95% CI)	−0.60 (−0.78 to −0.43)	−0.54 (−0.73 to −0.35)	−0.61 (−0.78 to −0.43)	−0.51 (−0.71 to −0.31)	−0.51 (−0.72 to −0.31)
	*p* < 0.001	*p* < 0.001	*p* < 0.001	*p* < 0.001	*p* < 0.001
DLQI change^c^ (95% CI)	−0.27 (−0.52 to −0.01)	−0.31 (−0.55 to −0.06)	−0.30 (−0.54 to −0.06)	−0.27 (−0.49 to −0.04)	−0.17 (−0.43 to −0.08)
	*p* = 0.019	*p* = 0.006	*p* = 0.007	*p* = 0.019	*p* = 0.127
Treatment sufficiency change, assessed by patient^d^ (95% CI)	0.63 (0.41 to 0.84)	0.55 (0.31 to 0.79)	0.59 (0.37 to 0.82)	0.38 (0.12 to 0.64)	0.71 (0.57 to 0.85)
	*p* < 0.001	*p* < 0.001	*p* < 0.001	*p* = 0.001	*p* < 0.001
Global impression of change in disease control at follow‐up, assessed by patient (pat‐GA‐control‐change)^e^ (95% CI)	−0.33 (−0.53 to −0.12)	−0.37 (−0.57 to −0.17)	−0.34 (−0.55 to −0.13)	−0.10 (−0.34 to 0.13)	−0.33 (−0.53 to −0.12)
	*p* = 0.004	*p* = 0.001	*p* = 0.003	*p* = 0.384	*p* = 0.005
Global disease control change, assessed by physician (change in phy‐GA‐control)^f^ (95% CI)	0.73 (0.52 to 0.93)	0.74 (0.55 to 0.94)	0.68 (0.45 to 0.91)	0.48 (0.18 to 0.76)	0.51 (0.21 to 0.80)
	*p* < 0.001	*p* < 0.001	*p* < 0.001	*p* = 0.003	*p* = 0.001
Treatment sufficiency change, assessed by physician^g^ (95% CI)	0.64 (0.40 to 0.88)	0.64 (0.45 to 0.84)	0.64 (0.39 to 0.89)	0.50 (0.21 to 0.79)	0.38 (−0.05 to 0.80)
	*p* < 0.001	*p* < 0.001	*p* < 0.001	*p* = 0.002	*p* = 0.024
Global impression of change in disease control at follow‐up, assessed by physician (phy‐GA‐control‐change)^h^ (95% CI)	−0.52 (−0.74 to −0.29)	−0.48 (−0.71 to −0.24)	−0.50 (−0.73 to −0.27)	−0.27 (−0.57 to 0.02)	−0.42 (−0.68 to −0.15)
	*p* < 0.001	*p* = 0.001	*p* < 0.001	*p* = 0.073	*p* = 0.005

*Note*: Presented are the results of rank correlation with the correlation coefficient Spearman's *rho*. A coefficient of 0.1–0.3 is considered a weak correlation, 0.3–0.5 a moderate correlation and >0.5 a high correlation. The correlation coefficient is negative in terms of the correlation of changes in the AECT with changes in AE‐QoL and DLQI, because higher AECT scores represent higher degree of disease control, whereas higher AE‐QoL and DLQI scores represent more impact on quality of life. Likewise, the Pat‐GA‐control‐change and Phy‐GA‐control‐change have negative correlation coefficients because positive scores represent improvement of disease control, which correlates with negative delta AECT scores (i.e. AECT score change = AECT at baseline—AECT at follow‐up). The first anchor was assessed by asking patients to self‐rate their global angioedema control during the past 4 weeks on a 5‐point Likert scale (answer options: ‘completely controlled’, ‘well controlled’, ‘moderately controlled’, ‘hardly controlled’, ‘not at all controlled’).

Abbreviations: AECT, Angioedema Control Test; AE‐QoL, Angioedema Quality of Life Questionnaire; CI, confidence interval; DLQI, Dermatology Life Quality Index; Q1‐4, Questions 1‐4 of the AECT.

^a^ *n* = 72.

^b^ *n* = 80.

^c^ *n* = 78.

^d^ *n* = 69.

^e^ *n* = 74.

^f^ *n* = 37.

^g^ *n* = 36.

^h^ *n* = 44.

For 18 cases, treatment changed from insufficient to sufficient, which was associated with a mean improvement in the AECT total score of 6.0 points. For three cases, treatment changed from sufficient to insufficient, which was associated with a mean deterioration in the AECT total score of 6.3 points. Treatment was and remained insufficient for 10 cases, associated with a mean change of 0.0 points in the AECT total score. Treatment was and remained sufficient for 37 cases, associated with a mean improvement of 1.0 point in the AECT total score.

### 3.3 The MCID of the AECT is three points

**FIGURE 2:**
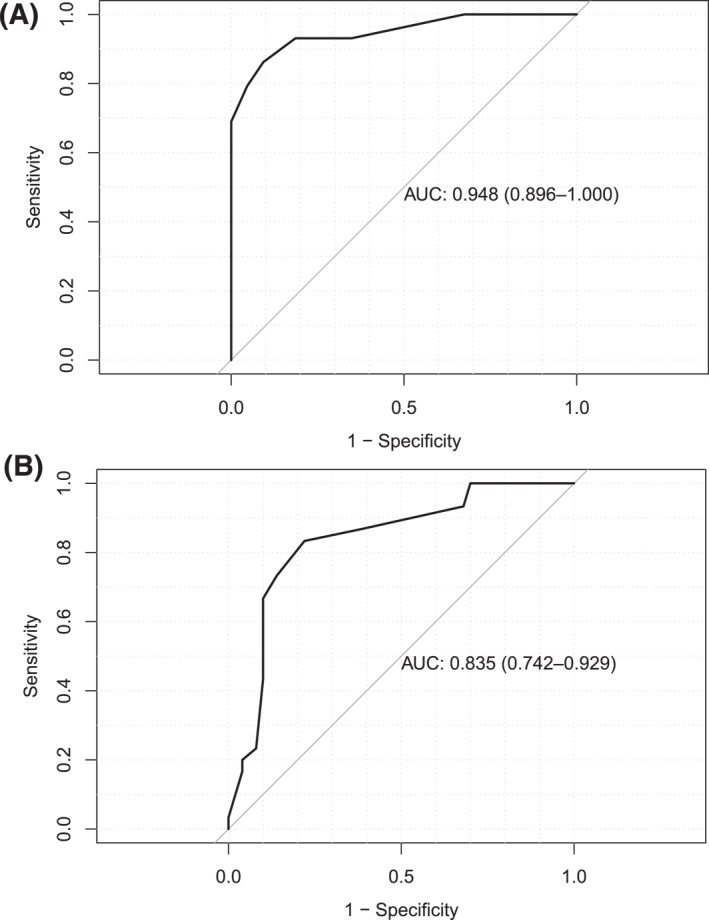
ROC curves. (A) Area under the ROC curve for AECT changes related to improvement versus non‐improvement in patients' self‐rated global angioedema control = 0.95 (95% confidence interval: 0.90–1.00). (B) Area under the ROC curve for AECT changes related to improvement versus non‐improvement in patients' self‐rated angioedema‐related quality of life = 0.96 (95% confidence interval: 0.89–1.00). AECT, Angioedema Control Test; ROC, receiver operating characteristic.

Four anchor‐based approaches were used to determine the MCID of the AECT. First (Table 3), for cases who reported relevant improvement of disease control, that is, one step in the Pat‐GA‐control, the mean (±SD) change in the AECT value was 4.5 ± 2.6 (median: 4.5 points, IQR: 2.8–6.0 points). Second, cases with a pertinent improvement in quality of life, that is, a one‐step (6–11 points) improvement in the AE‐QoL, showed a change in AECT of 2.9 ± 2.9 points (median: 3.0 points, IQR: 2.0–4.0 points). Third, by ROC curve analysis, the cut‐off point for AECT improvement with the best balance of sensitivity (86%) and specificity (91%) was found to be three points, based on the change in Pat‐GA‐control (Table 4, Figure 2A). Fourth, the change in AE‐QoL score, by ROC analysis, also supported an MCID of three points (Table 4, sensitivity: 73% and specificity: 86%, Figure 2B).

**TABLE 3:** Magnitude of AECT total and individual question score changes (mean ± SD) during improved or deteriorated angioedema control assessed by patients (e.g. one‐step change in global control means from not at all controlled to hardly controlled, completely controlled to well controlled, etc.) and improved or deteriorated AE‐QoL (one‐step = 6 to <12 points, two steps = 12 to <18 points).

	AECT total score change	AECT Q1 change	AECT Q2 change	AECT Q3 change	AECT Q4 change
Global control					
Improved global control by two steps	−6.222 ± 3.930	−1.556 ± 1.509	−1.556 ± 1.130	−1.111 ± 1.167	−2.000 ± 0.866
	Median: −6.000 *n* = 9	Median: −2.000 *n* = 9	Median: −1.000 *n* = 9	Median: −1.000 *n* = 9	Median: −2.000 *n* = 9
Improved global control by one step	−4.500 ± 2.646	−1.583 ± 1.443	−1.167 ± 0.577	−0.833 ± 0.937	−0.917 ± 0.515
	Median: −4.500 *n* = 12	Median: −1.000 *n* = 12	Median: −1.000 *n* = 12	Median: −0.500 *n* = 12	Median: −1.000 *n* = 12
Unchanged global control	−0.167 ± 2.091	−0.139 ± 0.899	0.056 ± 0.893	−0.056 ± 0.754	−0.028 ± 0.654
	Median: 0.000 *n* = 36	Median: 0.000 *n* = 36	Median: 0.000 *n* = 36	Median: 0.000 *n* = 36	Median: 0.000 *n* = 36
Deteriorated global control by one step	5.400 ± 1.673	1.600 ± 0.545	1.400 ± 0.545	1.200 ± 0.837	1.200 ± 0.447
	Median: 5.000 *n* = 5	Median: 2.000 *n* = 5	Median: 1.000 *n* = 5	Median: 1.000 *n* = 5	Median: 1.000 *n* = 5
Quality of life					
AE‐QoL improved with 12–18 points	−5.778 ± 2.386	−1.667 ± 1.118	−1.333 ± 0.707	−1.222 ± 0.667	−1.156 ± 1.509
	Median: −6.000 *n* = 9	Median: −2.000 *n* = 9	Median: −1.000 *n* = 9	Median: −1.000 *n* = 9	Median: −1.000 *n* = 9
AE‐QoL improved with 6–11 points	−2.923 ± 2.871	−0.923 ± 1.256	−0.846 ± 0.555	−0.308 ± 0.855	−0.846 ± 1.573
	Median: −3.000 *n* = 13	Median: −1.000 *n* = 13	Median: −1.000 *n* = 13	Median: 0.000 *n* = 13	Median: −1.000 *n* = 13
Unchanged AE‐QoL	−0.706 ± 3.030	−0.177 ± 0.999	−0.177 ± 0.936	−0.147 ± 0.744	−0.206 ± 0.770
	Median: 0.000 *n* = 34	Median: 0.000 *n* = 34	Median: 0.000 *n* = 34	Median: 0.000 *n* = 34	Median: 0.000 *n* = 34
AE‐QoL deteriorated with 6–11 points	1.667 ± 2.733	0.167 ± 0.753	0.167 ± 0.753	0.333 ± 1.033	1.000 ± 1.789
	Median: 1.500 *n* = 6	Median: 0.000 *n* = 6	Median: 0.000 *n* = 6	Median: 0.000 *n* = 6	Median: 0.500 *n* = 6
AE‐QoL deteriorated with 12–17 points	−0.167 ± 5.672	−0.333 ± 1.751	0.333 ± 1.633	0.667 ± 1.211	−0.833 ± 1.722
	Median: −1.000 *n* = 6	Median: −0.500 *n* = 6	Median: 0.500 *n* = 6	Median: 0.500 *n* = 6	Median: 0.000 *n* = 6

*Note*: The AE‐QoL score ranges were based on the MCID of the AE‐QoL, that is, 6 points.

Abbreviations: AECT, Angioedema Control Test; AE‐QoL, Angioedema Quality of Life questionnaire; SD, standard deviation; Q1‐4, Questions 1‐4 of the AECT.

**TABLE 4:** Performance of the AECT at various cut‐off values in screening for a meaningful improvement in global angioedema control and AE‐QoL (6 or more points).

	Global control			Quality of life		
AECT score improvement (cut‐off value)	Sensitivity (patients correctly classified as global control improved) (%)	Specificity (patients correctly classified as global control not improved) (%)	Cohen's kappa	Sensitivity (patients correctly classified as improved AE‐QoL) (%)	Specificity (patients correctly classified as not improved AE‐QoL) (%)	Cohen's kappa
0	29/29 (100.0)	14/43 (32.6)	0.28	28/30 (93.3)	16/50 (32.0)	0.21
1	27/29 (93.1)	28/43 (65.1)	0.54	26/30 (86.7)	31/50 (62.0)	0.44
2	27/29 (93.1)	35/43 (81.4)	0.72	25/30 (83.3)	39/50 (78.0)	0.59
3	25/29 (86.2)	39/43 (90.7)	0.77	22/30 (73.3)	43/50 (86.0)	0.60
4	23/29 (79.3)	41/43 (95.3)	0.76	20/30 (66.7)	45/50 (90.0)	0.59
5	20/29 (67.0)	43/43 (100.0)	0.73	16/30 (53.3)	45/50 (90.0)	0.46
6	18/29 (62.1)	43/43 (100.0)	0.66	13/30 (43.3)	45/50 (90.0)	0.36
7	11/29 (37.9)	43/43 (100.0)	0.42	7/30 (23.3)	46/50 (92.0)	0.18
8	8/29 (27.6)	43/43 (100.0)	0.31	6/30 (20.0)	48/50 (96.0)	0.19
9	7/29 (24.1)	43/43 (100.0)	0.28	5/30 (16.7)	48/50 (96.0)	0.15
10	1/29 (3.4)	43/43 (100.0)	0.04	1/30 (3.3)	50/50 (100.0)	0.04
11	1/29 (3.4)	43/43 (100.0)	0.04	1/30 (3.3)	50/50 (100.0)	0.04
12	1/29 (3.4)	43/43 (100.0)	0.04	1/30 (3.3)	50/50 (100.0)	0.04
13	1/29 (3.4)	43/43 (100.0)	0.04	1/30 (3.3)	50/50 (100.0)	0.04
14	1/29 (3.4)	43/43 (100.0)	0.04	1/30 (3.3)	50/50 (100.0)	0.04
15	1/29 (3.4)	43/43 (100.0)	0.04	1/30 (3.3)	50/50 (100.0)	0.04
16	0/29 (0.0)	43/43 (100.0)	0.00	0/30 (0.0)	50/50 (100.0)	0.00

Abbreviations: AECT, Angioedema Control Test; AE‐QoL, Angioedema Quality of Life questionnaire.

In addition, we calculated the MCID of the AECT by the use of two distributional criterion approaches. First, we divided the SD of all baseline AECT total score values (4.9) by two, which resulted in an MCID of 2.5 points. Second, the calculation of the SEM yielded an AECT MCID of 0.5 points.

### 3.4 Sensitivity analyses

Repeating the analyses described above using only data from unique individuals (*n* = 66) resulted in comparable correlation coefficients and MCID estimates (data not shown).

## 4 DISCUSSION

Here we report, for the first time, sensitivity to change and responsiveness of the AECT as well as its MCID. The AECT is an easy to use and validated PROM developed to assess disease control in patients with recurrent angioedema.^18^ It is available in many languages^36^ and widely used in clinical trials and routine practice as recommended by current international guidelines.^6^, ^8^

We found that changes in the AECT correlate well with anchor instruments that measure changes in angioedema control, health‐related QoL, and treatment sufficiency. However, the correlation of a change in the total AECT score with a patients' global impression of change in angioedema control assessed at the follow‐up visit was lower than expected. We believe this is due to recall bias, an assumption strengthened by the observation that, in contrast, a good correlation between changes in the AECT score and changes in the patients' assessed global angioedema control was found.

The availability of an MCID is critical for the interpretation of results obtained by a PROM. By applying anchor‐based and distributional criterion‐based approaches, we found the MCID of the AECT to be between 2.5 and 4.5 points. We clearly favour the mean‐change and ROC curve analysis anchor‐based approaches over the distributional‐based approaches, since they represent more direct and patient‐centred methods and are generally accepted to have higher clinical relevance. Therefore, we recommend three points to be used as the MCID for the AECT score for improvement in angioedema control. In other words, an increase in the AECT score by three points or more can be regarded as a meaningful change to the patient.

A limitation of our study is the low number of cases experiencing deteriorating disease control in our sample. Thus, the available data do not allow for the computation of the MCID for deterioration. The MCID for improvement (three points) cannot simply be applied to deterioration, since it is known that these often differ. A meta‐analysis of 118 prospective cohort studies showed that generally smaller estimates for improvement compared with deterioration are found.^37^ Furthermore, the cause of recurrent angioedema was unknown in three patients included in this study. Still, they did experience recurrent angioedema and the AECT was thus an applicable tool to measure their disease control, even if the underlying pathophysiology was not fully clarified at the time the patients took part and completed the questionnaires. Another limitation of this study is the scarcity of available data needed for stratification based on baseline angioedema control (i.e. dichotomisation in patients with poorly and well‐controlled angioedema) or type of angioedema. We cannot fully exclude that the MCID of the AECT may differ based on how well the disease is controlled when the AECT is filled‐out for the first time. Likewise, we cannot fully exclude that the MCID differs for patients with HAE‐C1INH as compared to patients with mast cell‐mediated angioedema. More than half (59%) of the participants had bradykinin‐mediated angioedema, with HAE‐C1INH as the most frequent diagnosis (41%). Therefore, our results may translate better to patients with bradykinin‐mediated than mast cell‐mediated angioedema. Further studies including more participants are required to provide definite answers.

In conclusion, the AECT is a valuable tool to measure levels and changes of angioedema control in patients with recurrent angioedema, and is thus a suitable tool for assessing treatment responses. The knowledge of the MCID of three points for improvement increases the interpretability of AECT results and further recommends its use in clinical trials and routine patient care.

## AUTHOR CONTRIBUTIONS

All authors made substantial contributions to (i) conception and design of or acquisition of data or analysis and interpretation of data, to (ii) drafting the article or revising it critically for important intellectual content and to (iii) final approval of the version to be published.

## CONFLICT OF INTEREST STATEMENT

KW, MM and MM are advisors for Moxie. All other authors have no conflict of interest regarding this manuscript.

## Data Availability

The data that support the findings of this study are available from the corresponding author upon reasonable request.
